# Quackery in Dental Practice in Nepal

**DOI:** 10.31729/jnma.5036

**Published:** 2020-07-31

**Authors:** Manoj Humagain, Bishwa Prakash Bhattarai, Dinesh Rokaya

**Affiliations:** 1 Department of Periodontology, Kathmandu University School of Medical Sciences, Dhulikhel, Nepal; 2 Department of Oral and Maxillofacial Surgery, International College of Dentistry Walailak University, Bangkok, Thailand; 3 Department of Clinical Dentistry, International College of Dentistry Walailak University, Bangkok, Thailand

**Keywords:** *dental general practice*, *dentistry*, *ethics*, *Nepal*, *prosthetic dentistry*

## Abstract

Quackery and fraud in dental practice, seen in many countries, is also rampant in Nepal, and they are unethical practices. There is a growing need for strict enforcement of government policy measures to eliminate quackery and fraudulent dental practice in Nepal. The government should mobilize all dental workforce (dental specialists, dentists, and dental auxiliaries) and aware of their responsibilities and limitations. This article presents a brief review showing some cases of malpractice in dentistry in Nepal.

## INTRODUCTION

The dentist per population ratio of Nepal is 1:20000, which is almost three folds less than the recommended ratio by the World Health Organization (WHO).^1^ Legally, the dental practice in Nepal can be conducted by a registered dentist or under their supervision.^2^ Quackery, charlatanism, fraud, incompetence, and any other malpractice that jeopardizes the health of the dental patient should be opposed.^3^ Quackery and fraud in dental practice is rampant in Nepal, crossing the whole strata of registered dental practitioners.^4–7^ Furthermore, academic credentials of several dentists have come under government scrutiny for forgery, due to reports on unethical dental and medical malpractice.^8^

## QUACKERY IN NEPAL

However, quackery is rooted in both urban and rural areas, including the capital city, where over 600 dental clinics are run by dental hygienists alone who are not permitted to practice full-fledged dentistry. Quackery poses a big threat to the integrity of the dental profession and the patients in Nepal and has proved to be a big hurdle to overcome. Although the government has laid out strict policies pertaining to the dental clinic and dental hospital setup requirements such as the number of dental units, types and number of oral health care professionals (dental specialist, dentist, dental hygienist, and assistant), and sterilization protocols including management strategies,^9^ most dental clinics and hospital setups in Nepal do not comply to these rules.

### COMMON UNETHICAL PRACTICES AND MALPRACTICES

In Nepal, various dental specialties, and have their own unethical dental treatments and their consequences (Table 1).

**Table 1:** Common unethical dental practice and their consequences in various areas in Nepal.

Areas	Common unethical dental practices/ malpractices	Consequences
General	Improper clinic hygiene Improper sterilization of the instruments	Infections Transmission of diseases
Restorative Dentistry	Failure to diagnose caries Inadequate caries removal Over cavity preparations and destruction of teeth Improper section of restorative materials Improper restorations	Recurrent caries Failure of restorations Teeth fracture
Endodontics	Failure to locate canals Improper cleaning and shaping of canals Improper root canal treatment (RCT)	Apical perforation, ledge formation, and transportations Root canal treatment failure Teeth fracture
Prosthodontics	Fixing removable dentures Faulty self-cure acrylic crowns Self-cure bridges Faulty removable dentures	Compromise oral hygiene Short and long-term effects from the toxic materials, i.e., burning, toxicity, and carcinogenicity. Toxicities from the metal prosthesis, i.e. Ni, Cr, Al, etc.
Oral and maxillofacial surgery	Painful extraction Improper extraction Fracture of teeth and/or root during teeth extraction Prolonged bleeding Incorrect diagnosis of oral lesions Improper prescription of antibiotics.	Retained roots Damage to surrounding structures. Prolonged bleeding leading to syncope, hospitalization, or and/or death. Small lesions can progress to large lesions. No effect or adverse effects of antibiotics Antibiotic resistance
Orthodontics	Braces done by an unqualified person.	Improper teeth movement. Prolong treatment. Compromise oral hygiene.

**Figure 1.**
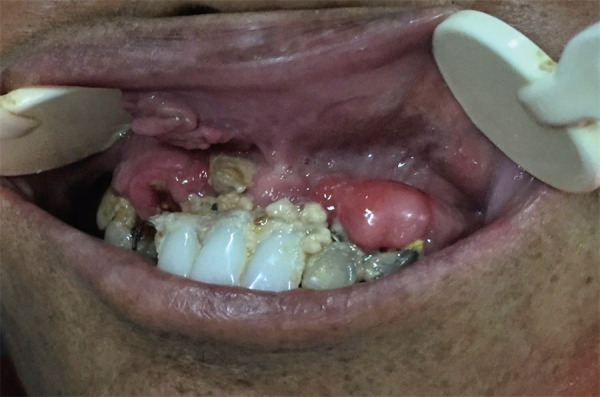
Faulty prosthetic restoration (fixed-removable partial denture) and improper oral hygiene leading to the destruction of the soft and hard oral structures.

The most common unethical dental procedures include fixing removable dentures, faulty self-cure acrylic crowns, self-cure bridges, improper root canal treatments (RCT), etc. Incorrect prosthetic restoration results in inadequate hygiene and destruction of the soft tissue and hard tissues of the oral structures (Figure 1).

**Figure 2.**
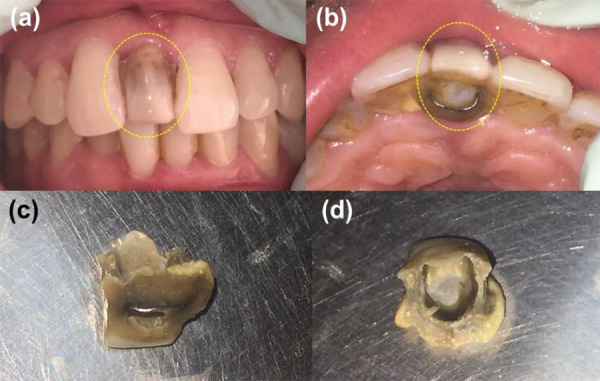
Fixed-removable denture; front view (a) and occlusal view (b) in the patient's mouth and the palatal view side (c) and inside view (b) of the fixed crown.

The fixed-removable denture is shown below (Figure 2). The self-cure acrylic crown was fixed with adjacent teeth using stainless steel wire and self-cure acrylic reason. This case was seen in early after the delivery of the prosthesis; hence the effects on gingiva were less.

**Figure 3.**
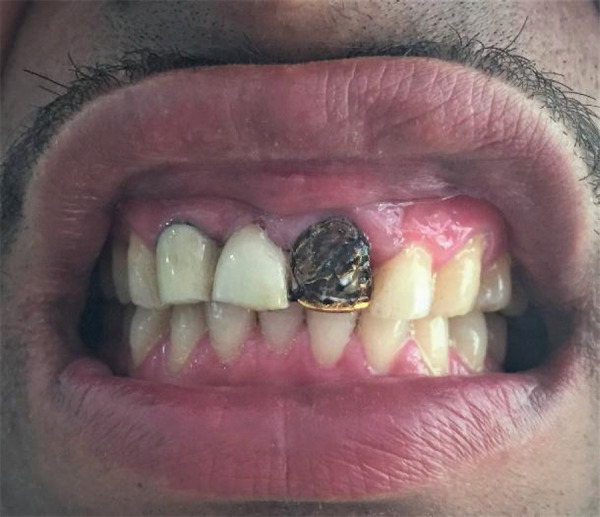
A faulty metal crown on the maxillary left central incisor with the detached acrylic cover on the labial surface.

A faulty metal crown on the maxillary left central incisor with the detached acrylic cover on the labial surface is shown in Figure 3. It showed that previously acrylic veneer was attached on the labial surface and is detached, causing anesthetic teeth.

Hence, unethical dental practice done by unqualified personnel is seen in Kathmandu and other cities.^10^ Performing dental treatments beyond the limit of their education, knowledge, and practice is unethical and such practices should be prohibited. Nepal Medical Council (NMC) has developed a code of ethics for its registered dental doctors for ethical dental practice.^11,12^

## WAY FORWARD

There is a growing need for increased vigilance and strict enforcement of government policy measures to eliminate quackery and fraudulent dental practice in Nepal. Constant inspections and closure of dental clinics and hospital which do not meet the criteria will help to discourage quackery and promote standard ethical practice. An adequate number of quality oral health care professionals can be produced and mobilized throughout the country by the government in co-operation with dental academic institutions.^2^ However, at present, about 85% of the dentists are concentrated in the capital city, while government positions for dentists are limited to zonal (provincial) hospitals. More jobs are needed for dentists at district hospitals and primary healthcare centers. Mobilization of the dental workforce (dental specialist, dentist, and dental auxiliaries) and making aware of their responsibilities and limitations, making affordable dental care services in government hospitals and dental insurance system maybe some of the solutions for the eliminations of the quackery and unethical practice in Nepal.

Finally, providing affordable and accessible dental services, awareness in people to choose registered dental practitioners over quacks. The government should also include dental treatments in the universal coverage system. Until which, elimination of dental quackery in Nepal seems a goal hard to attain.

There is a growing need for increased vigilance and strict enforcement of government policy measures to eliminate quackery and fraudulent dental practice in Nepal. The quackery and fraud in dental practice are unethical dental practices, and they should be stopped. Mobilization of dental workforce (dental specialists, dentists, and dental auxiliaries)and making aware of their responsibilities and limitations, making affordable dental care services in government hospitals and provision of dental insurance system maybe some of the solutions for the eliminations of the quackery and unethical practice in Nepal.

## Conflict of Interest

**None.**
